# An Unusual Case of Extensively Drug Resistant Typhoid Fever

**DOI:** 10.7759/cureus.4664

**Published:** 2019-05-14

**Authors:** Jamil M Bhatti, Yousuf Memon, Samreen Sarfaraz, Naseem Salahuddin

**Affiliations:** 1 Infectious Diseases, The Indus Hospital, Karachi, PAK; 2 Radiology, The Indus Hospital, Karachi, PAK

**Keywords:** xdr typhoid, salmonella typhi, hepatitis, disseminated intravascular coagulation, meropenem, azithromycin

## Abstract

Enteric fever is a potentially fatal multisystemic illness caused primarily by *Salmonella typhi *and, to a lesser extent, by Paratyphi A, B, and C. Emergence of resistance has depleted the antimicrobial arsenal overtime, making treatment challenging and costly. In 2016, a new extensively drug resistant (XDR) strain of *Salmonella typhi *emerged in Sindh, which only responds to two antibiotics--carbapenems and azithromycin. Its clinical spectrum is not yet clear but increased morbidity and mortality is being observed with it. We present a severe case of XDR *Salmonella typhi* where the clinical course was complicated by delayed defervescence, severe hepatitis, soft tissue infection, and profuse lower gastrointestinal bleeding, which responded to a combination of carbapenem and azithromycin and an invasive procedure to contain bleeding from the cecal artery. The purpose of this case report is to highlight the morbidity, cost, and therapeutic challenges associated with severe XDR *Salmonella typhi* infection.

## Introduction

Enteric fever by *S. typhi* can result in severe disease with complications in 10%-15% of patients, including gastrointestinal bleeding, intestinal perforation, hepatitis, pancreatitis, typhoid encephalopathy, disseminated intravascular coagulation, hemolytic uremic syndrome, endocarditis, pneumonia, and, rarely, reactive hemophagocytic lymphohistiocytosis (HLH) and rhabdomyolysis [1,2].

Host factors associated with severe disease include infants, immunosuppression, female sex, and those infected with drug-resistant *S. typhi* [2]. Specific genetic polymorphisms in major histocompatibility complex class II and III genes have also been found in individuals with severe typhoid fever.

The bacterium grows intracellularly in the intestinal lymphoid tissue, presenting acutely with gastrointestinal symptoms and fever. The classic presentation is that of a prolonged fever, step-ladder in pattern, accompanied by malaise, abdominal pain, and constipation in the first two weeks, followed by diarrhea in the third week [3]. Metastatic infection in the liver, spleen, bone, and other viscera may occur. Many patients develop gastrointestinal bleeding and intestinal perforation.

Typhoid fever is a serious water-borne infection caused by *Salmonella enterica *that remains endemic in Pakistan due to contaminated water supplies and deplorable sanitary conditions. Over a 100,000 cases are reported annually. The emergence of drug-resistant strains has depleted the antimicrobial arsenal overtime, making treatment challenging and costly. In 2016, a new extensively drug resistant (XDR) strain resistant to five antibiotics emerged in Hyderabad and spread, infecting more than 8,000 people in 14 districts across Sindh up till now. Due to the acquisition of H58 haplotype mutation it does not respond to third generation cephalosporins. Carbapenems and azithromycin are antibiotics of last resort and further mutation threatens to make typhoid untreatable. Disturbing reports of azithromycin resistance are already emerging from South East Asia. The clinical spectrum of XDR *Salmonella* infection is unclear and it may increase the morbidity and mortality associated with typhoid.

## Case presentation

A 31-year-old woman was admitted with high grade intermittent fever, jaundice, and watery diarrhea of one month. A week into the febrile illness, she developed pain and inflammation over the right upper arm extending below the elbow. Her past history was relevant only for controlled hypothyroidism.

On examination, the patient was alert, oriented, but ill-appearing with fever, dehydration, and icterus. There was an inflamed, indurated swelling with blisters extending from the distal third of the upper arm to the proximal one-third of the forearm, involving the elbow joint. Movement at the joint was limited due to pain. The remainder of the examination was unremarkable except for mild tenderness over the epigastrium. Her laboratory investigations are shown in Table 1.

**Table 1:** Laboratory data *abnormal values

Variables	Reference Range	On Admission	Peak Values
Hemoglobin (g/dl)	13.7-16.3	12.2	
PCV (Packed Cell Volume)	41.9-48.7	39.5	
White Cell Count (per mm3)	4,000-10,000	9680	
Differential Count %			
Neutrophils	40-70%	51.1%	
Lymphocytes	20-45%	38.8%	
Monocytes	02-10%	7%	
Eosinophils	01-06%	2.3%	
Basophils	00-01%	0.8%	
Platelets	150-400*109	319	
Prothrombin Time (sec)	11-16sec	14sec	21 sec (INR 1.85)*
Creatinine (mg/dl)	0.7-1.2	0.65	
Potassium (mEq/L)	3.5-5.0	4.48	
Serum Albumin (g/dl)	3.8-4.4	3.06	
Total Bilirubin (mg/dl)	0-1.4	2.49*	4.94*
Direct Bilirubin (mg/dl)	0-0.3	2.02*	
GGT (Gamma Glutamate Dehydrogenase) (U/L)	08-61	425*	
ALT(Alanine Aminotransferase) (mg/dl)	0-41	123*	
Alkaline phosphatase (mg/dl)	40-129	211*	

Viral hepatitis and human immunodeficiency virus (HIV) antibodies were negative. Two blood cultures grew *Salmonella typhi *that was resistant to ampicillin, chloramphenicol, co-trimoxazole, ciprofloxacin, ceftriaxone and cefixime, and sensitive to all carbapenems and azithromycin. An ultrasound (US) of the elbow revealed large fluid collection with septations, which was incised and drained. *Salmonella typhi *was isolated from culture of the pus with identical sensitivities as in the blood cultures.

**Figure 1:**
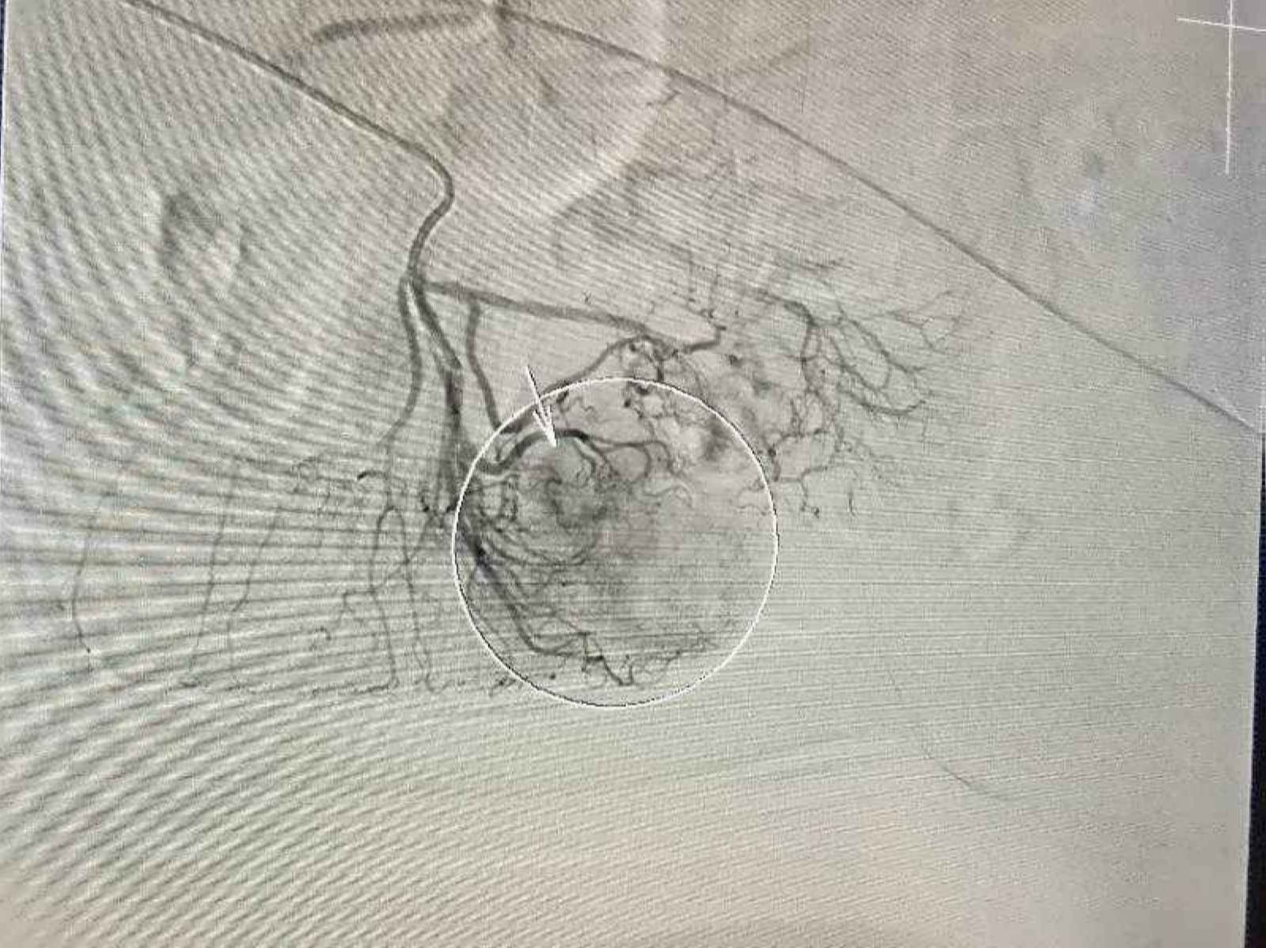
Image shows blush of the contrast (arrow and circle) from the cecal artery indicating acute bleeding in the cecum

Meropenem 1 gm i/v 8 hourly and azithromycin 500 mg i/v od were started intravenously, but she continued to have high fever and the dose of meropenem was doubled on Day 5. During admission, the patient developed severe epigastric pain and vomiting for which she was kept nil per oral and a nasogastric tube was placed. She started to have small episodes of per rectal bleed from the eighth day of illness leading to an intermittent drop in hemoglobin. D-dimers were >15000 and fibrin degradation products >20 ug/ml suggestive of disseminated intravascular coagulation (DIC), although the activated partial thromboplastin time (APTT) remained within normal limits. Supportive blood and blood products were continued. On the 11th day the patient started having massive per rectal bleed, became hypotensive and remained so despite aggressive blood transfusions. A computed tomography (CT) scan of the abdomen reported thickening at the ileocecal junction and cecal pole with significant mesenteric lymphadenopathy and fibrotic bands in both lung fields. Digital subtraction angiography was done to identify the source of gastrointestinal (GI) bleeding. Angiography of the superior mesenteric artery (SMA), inferior mesenteric artery (IMA), mesenteric arteries, and celiac artery was performed using a 5F catheter and 035 hydrophilic guide wire. Non-ionic contrast was injected. Bleeding was detected from the cecal branch of the SMA, seen as a blush. Figure 1 shows blush of the contrast (arrow and circle) from the cecal artery, indicating acute bleeding in the cecum.

**Figure 2:**
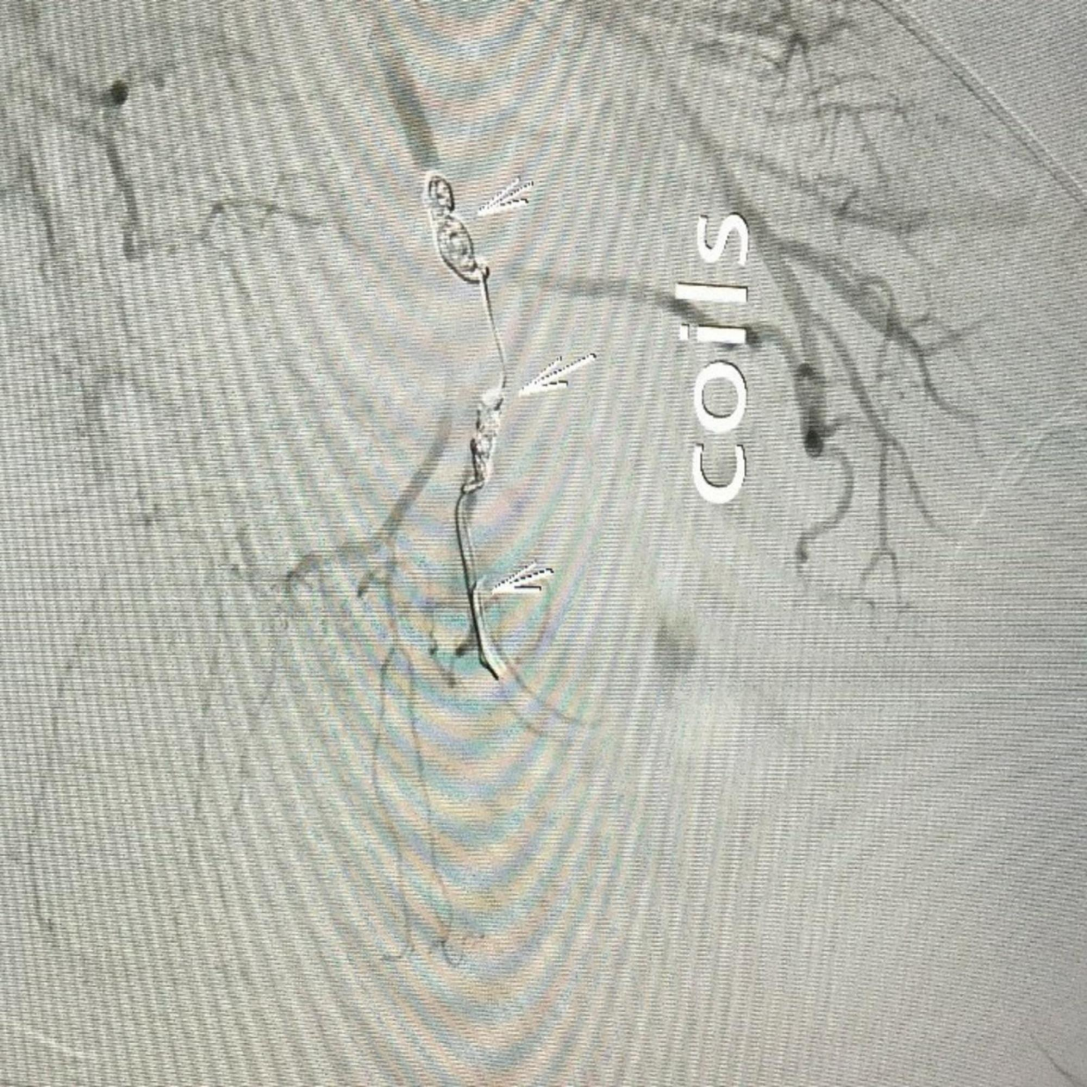
Post embolization by coils (arrows) and polyvinyl alcohol (PVA) particles, showing cessation of contrast extravasation

The bleeding was stopped by using micro coils. The final angiogram showed cessation of bleeding (Figure 2).

Defervescence occurred on Day 12. The patient subsequently improved clinically and her lab parameters returned to normal. She was discharged to home care on azithromycin 500 mg daily for an additional one week. Follow-up as an outpatient an additional two weeks later showed complete recovery from the infection.

## Discussion

Our patient had XDR *S. typhi* strain that was resistant to five classes of antibiotics, i.e. chloramphenicol, ampicillin, trimethoprim-sulfamethoxazole, fluoroquinolones, and third-generation cephalosporin. Although outbreaks of XDR *S. typhi* have been seen in different parts of Pakistan, its prevalence is rare in the Western world [1,2].

Our patient had several unique features. Ileocecal thickening, as seen in our patient, could have been due to neoplastic, inflammatory, infectious or ischemic conditions but also be a normal variant [3]. In general, benign conditions result in bowel wall thickening of less than 2 cm, whereas wall thickening greater than 3 cm usually indicates a neoplastic condition. However, entities that cause mild bowel wall thickening often overlap, and wall thickening may be marked in infectious or ischemic processes. Ileocecal thickening is a rare complication of typhoid infection as seen in our patient but it is well published in literature [4]. Mesenteric lymphadenopathy is more common in nontyphoidal salmonellosis [5].

Superficial skin abscesses are an unusual presentation of *S. typhi *septicemia, most notably occurring where there is an underlying predisposition, such as sickle cell anemia and in severely ill or immunocompromised patients [6-9]. In a series of 240 adult cases of typhoid fever, subcutaneous abscesses from which *S. typhi* was isolated, occurred in 2.9% of patients having an underlying predisposing condition [10]. Our patient is unique in that the large subcutaneous abscess occurred without any underlying risk factor.

Typhoidal hepatitis with transaminitis occurs in 21%-60% of cases of typhoid fever [11,12]. However, severe hepatic derangement simulating acute viral hepatitis is very rare. The presentation with elevation of transaminases, total bilirubin, gamma glutamate dehydrogenase (GGT) and deranged prothrombin time/ international normalized ratio (PT/INR) similar to that of severe acute viral hepatitis, as observed in this case, makes this case unique. Cases of acute viral hepatitis with typhoid have been reported by others [13-15].

Although mild gastrointestinal bleeding is common in enteric fever, massive, lower gastrointestinal bleeding is rare. The terminal ileum is the most commonly involved site, followed by the ileocecal valve, ascending colon, and transverse colon [16]. There are case reports of enteric fever presenting with massive gastrointestinal hemorrhage requiring CT angiography with a view towards embolization or endoscopic hemoclipping [17,18].

Fever defervescence time in multidrug resistant (MDR) typhoid ranges from 3.9 to 5.7 days as reported by Jeeyani et al. [19]. A six-year retrospective survey from a tertiary care hospital in Singapore also found that the median value for time from initiation of appropriate antibiotic treatment to fever defervescence was five days [20]. There is no data available for time to fever defervescence in XDR *Salmonella.*

This case had several unusual features: XDR *Salmonella typhi* complicated by acute hepatitis, large soft tissue abscesses, DIC, CT scan evidence of thickened ileocecal junction with significant mesenteric lymphadenopathy, and massive lower GI bleeding that required invasive radiological intervention to stem the bleeding.

## Conclusions

Typhoid fever usually manifests with prolonged fever, transaminitis, and GI symptoms. The clinician must, however, be aware of diverse manifestations that mimic other conditions. Diagnosis of complicated typhoid is clinched only through isolation of *S. typhi *in suspected clinical specimens. In this case we were able to isolate an extensively drug resistant *S. typhi*. Appropriate selection of antibiotics was made only after identification of the bacteria and the drug sensitivity.
